# A systematic review of the role of clozapine for severe borderline personality disorder

**DOI:** 10.1007/s00213-023-06431-6

**Published:** 2023-08-12

**Authors:** Joshua Han, Stephen Allison, Jeffrey C.L. Looi, Sherry Kit Wa Chan, Tarun Bastiampillai

**Affiliations:** 1 College of Medicine and Public Health, Flinders University, Adelaide, South Australia Australia; 2 Discipline of Psychiatry, College of Medicine and Public Health, Flinders University, Adelaide, South Australia Australia; 3 Consortrium of Australian-Academic Psychiatrists for Independent Policy and Research Analysis (CAPIPRA), ACT Canberra, Australia; 4 Academic Unit of Psychiatry and Addiction Medicine, The Australian National University School of Medicine and Psychology, Canberra Hospital, Canberra, ACT Australia; 5 Department of Psychiatry, the School of Clinical Medicine, The University of Hong Kong, Hong Kong, Hong Kong; 6 The State Key Laboratory of Brain and Cognitive Sciences, The University of Hong Kong, Hong Kong, Hong Kong; 7 Department of Psychiatry, Monash University, Wellington Road, Clayton, Victoria Australia

**Keywords:** Clozapine, Severe borderline personality disorder, · Suicide, Recurrent self-harm

## Abstract

**Rationale:**

Clozapine is a unique medication with a potential role in the treatment of severe borderline personality disorder (BPD).

**Objectives:**

The review examines the effectiveness of clozapine as a medication for management for severe BPD with high risk of suicide, violence or imprisonment, and aims to help guide clinical practice in managing severe BPD.

**Methods:**

A database search of the terms “Clozapine” AND “BPD”; “Antipsychotics” AND “BPD”; “Clozapine” AND “Borderline Personality Disorder”; and “Antipsychotics” AND “Borderline Personality Disorder” were performed in CINAHL, Cochrane Library, Embase, Medline, PsychINFO, PubMed, and Web of Science. Full-text articles of clinical clozapine use for BPD were included for review.

**Results:**

A total of 24 articles consisting of 1 randomised control trial, 10 non-controlled trials, and 13 case reports were identified. Most of the studies reported benefits from clozapine when used for severe BPD. Many of the studies focused on clozapine use in BPD patients at high risk of suicide. Results from these non-controlled and case reports support the use of clozapine in patients with severe BPD at high risk of suicide.

**Conclusion:**

There may be a role for clozapine in treating severe treatment refractory BPD, especially for those patients at high risk of suicide and frequent hospitalisations.

## Introduction

Borderline personality disorder (BPD) is a cluster B personality disorder characterised by pervasive pattern of instability of self-image, interpersonal relationships, and affect, as well as poor impulse control (American Psychiatric Association 2013). Key complications of BPD include recurrent self-harm and suicide (Pompili et al. 2005). It is estimated up to 10% of patients with BPD die by suicide (Lieb et al. 2004). The main mode of treatment for BPD has been psychological rather than pharmacological (Bateman et al. 2015), due to insufficient evidence to support the latter (Bateman et al. 2015, Stoffers-Winterling et al. 2020). As such, current BPD treatment guidelines discourage the use of pharmacotherapy unless in significant crisis (Pascual et al. 2010, National Institute for Health and Care Excellence 2015). Despite the lack of evidence, pharmacotherapy remains widely used to assist people with personality disorders for symptomatic control (Stoffers-Winterling et al. 2020, Stoffers et al. 2010) and up to 90–95% of patients with BPD receive pharmacotherapy (Riffer et al. 2019, Bridler et al. 2015).

Pharmacological treatment for BPD has been investigated since 1981 (Soloff 1981). Antipsychotics are one of the main classes of drugs investigated for use in BPD (Abraham and Calabrese 2008). The use of antipsychotics for BPD remains common, with quetiapine, risperidone, and olanzapine being regularly used for the condition (Bridler et al. 2015, Hardoon et al. 2022). The use of clozapine for BPD, on the other hand, is uncommon compared to other antipsychotics (Bridler et al. 2015, Hardoon et al. 2022).

Clozapine is an atypical antipsychotic, well-known for its use in treatment-resistant schizophrenia (Kane et al. 1988, Meltzer 1997, Mortimer et al. 2010). However, it is associated with significant side effects, including myocarditis, constipation, sedation, hypersalivation, and weight gain (Miller 2000, Safferman et al. 1991), and the small risk of agranulocytosis (Miller 2000, Safferman et al. 1991, Alvir et al. 1993, Idänpään-Heikkilä et al. 1977, Wiciński and Węclewicz 2018).

Clozapine amongst antipsychotic medication uniquely reduces the risk of suicide in people with schizophrenia (Meltzer et al. 2003, Hennen and Baldessarini 2005, Zalsman et al. 2016, Chan et al. 2021). Due to these properties, clozapine is the only drug currently used specifically to reduce suicide risk (Keepers et al. 2020) and is the only drug approved for treating suicidality in the setting of schizophrenia by the FDA (U.S. Food and Drug Administration 2021). Lithium, long considered to reduce suicide and suicidal behaviour (Del Matto et al. 2020), has not been found effective in this capacity in a recent meta-analysis of randomised controlled trials (Nabi et al. 2022). The mechanism behind the anti-suicidal effects of clozapine is poorly understood but may be related to its effect on reducing impulsivity (Khokhar et al. 2018). Due to this unique property of clozapine in reducing suicide risk in schizophrenia, further research into its potential for the reduction of self-harm and suicide risk in BPD is warranted. In 2014, a non-systematic narrative review of evidence for the use of clozapine in those with BPD was conducted by Beri and Boydell (2014). This review highlighted the lack of an RCT or other higher quality comparative study regarding the use of clozapine in BPD. A systematic review was performed by Hancock-Johnson et al. (2017) reviewing all forms of pharmacological treatment for BPD which did not find any interventional or cohort studies investigating the role of clozapine treatment for BPD.

This systematic review investigates current evidence around the efficacy of clozapine as a medication for severe BPD patients at high risk of major adverse outcomes such as suicide, recurrent self-harm, and the use of hospitalisation. The secondary objective is to examine the optimal clozapine dose to achieve these desired effects in BPD, and to assess if clozapine has efficacy in specific domains of BPD.

## Method

The search terms used to locate articles were “Clozapine AND BPD”; “Antipsychotics AND BPD”; “Clozapine AND Borderline Personality Disorder”; and “Antipsychotics AND Borderline Personality Disorder.” No filters or limits were used for any of the searches. The decision for using broad terms was made to ensure that all relevant articles could be located, as a low number of relevant articles was anticipated. The databases searched were CINAHL, Cochrane Library, Embase, Medline, PsychINFO, PubMed, and Web of Science. These databases were last consulted in May 2021. The results from the database search were imported to Covidence which was then used to conduct the remainder of the review. Duplicate studies were then removed using Covidence’s inbuilt duplicate removal function. Remaining articles were then checked manually to identify and remove any duplicates that was missed by the inbuilt duplicate identification system. One author performed the title and abstract screening. Two of the authors independently screened the full texts of articles that were not excluded from the title and abstract screening. In case of conflict between authors, a consensus was reached through a discussion between the two authors. The reference list of retrieved papers was screened to identify any other possible articles that met the inclusion criteria. In addition, a search using the same terms were conducted on Google Scholar by one of the authors to identify any missed literature. The protocol for the systematic review was registered on PROSPERO with the registration ID of CRD42021248496.

### Inclusion and exclusion criteria

We included studies on patients with established diagnosis of BPD who were treated with clozapine. All studies where the target demographic were people with BPD was included even if the primary target condition, or symptomatology, was not BPD. Studies where the patient population had a concurrent psychiatric condition classically associated with psychosis were excluded, such as schizophrenia, schizophreniform disorders, drug-induced psychosis, and bipolar disorder.

### Synthesis

The studies were grouped into “Clozapine vs placebo RCT,” “Effect of clozapine pre- and post- treatment within the same patient,” and “case reports” groups. Data extraction was performed by one of the authors, which was then checked by another author for quality assurance. As most studies consisted of non-randomised non-controlled trials, case series, and case reports, the decision was made to not perform statistical analyses. Data collection included the number of participants, gender, reason for clozapine treatment, other treatments tried, outcomes measured, results (including statistics where statistical analysis was performed), dose of clozapine used, and adverse effects were extracted from the selected studies.

### Risk of Bias Assessment

Due to the presence of both randomised control trials (RCT) and non-randomised interventions within the extracted articles, two different risk of bias assessment tools were used. For risk assessment, case series were considered a non-randomised intervention.

Risk of bias assessments were performed using the Cochrane RoB 2.0 tool (Higgins et al. 2011) for RCT and Risk of Bias in Non-randomised Studies and Interventions (ROBINS-I) Tool (Sterne et al. 2016) for non-randomised studies of interventions. For the RoB 2.0 tool, the current version (22 August 2019) was used instead of the newer test version as the 2019 version was the latest validated version.

Due to the lack of consensus-based risk of bias assessment tool for case reports, a risk of bias assessment was not performed for these types of studies. Instead, the CAse REport (CARE) guidelines checklist (Gagnier et al. 2013) was used to assess the quality of these studies without directly assessing risk of bias. The guideline was originally developed as a reference for writing quality case reports. This review utilised this tool to assess the quality of published case reports as a checklist. This was achieved by determining the number of domains on the guideline that was satisfied by each report. In domains with subcategories, if the majority of the subcategories were satisfied, the domain was deemed as “satisfactory” and vice versa. In cases of equal number of subcategories in a domain being “satisfactory” and “unsatisfactory,” the item was scored as “equivocal.”

### Reporting

The Preferred Reporting Items for Systematic Reviews and Meta-Analyses (PRISMA) guidelines by Moher et al. (2009) and the updated PRISMA 2020 version (Page et al. 2021) were used as a reference for the reporting phase.

## Results

**Fig. 1:**
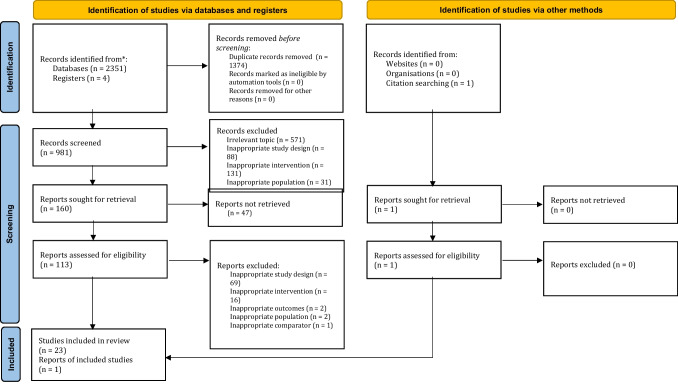
PRISMA flow diagram of included results

The database search yielded a total of 2355 articles. Of these, 1374 were duplicates, which resulted in net 981 articles that met the criteria. Another 821 articles were excluded after the title and abstract review. Of the remaining 160 articles, 23 relevant articles fit the inclusion criteria. After screening the reference list of the included articles, a further one article was determined to fit the inclusion criteria. In total, 24 articles were included in this review (Fig. 1).

Of the 24 included articles, one study was an RCT that failed to recruit the required number of participants, 7 were cohort studies without concurrent control groups, two were open-label trials, two were case series, and 12 were case reports. All but one study had low number of participants (less than 30). The only study with more than 30 participants was by Rohde et al. (2018), which was a registry-based study.

Only one out of the 12 non-case reports compared the efficacy of clozapine to another intervention (which was a placebo). The other 11 studies compared the pre- and post-clozapine treatment status within the same patients.

## Characteristics of studies

### Clozapine vs non-clozapine intervention

There was one RCT comparing clozapine with placebo (Crawford et al. 2022, Table 1). The primary outcome measure for the Crawford et al. study was the Zanarini Rating Scale for Borderline Personality Disorder (ZAN-BPD), which measures the severity of overall BPD symptoms (Zanarini 2003). The study failed to recruit the calculated required number of participants and had to be terminated early due to the COVID-19 pandemic. Nevertheless, a statistical analysis of the recruited participants for the duration of the study was performed. The study found better overall ZAN-BPD scores in the clozapine arm, but the result was not statistically significant compared to placebo. The study did acknowledge the low sample size and the low adherence rate in the clozapine arm as significant limitations. The main adverse drug effects noted during the study were gastrointestinal (including constipation), sedation, and cardiac (including tachycardia).

**Table 1:** Summary of studies (randomised/non-randomised) and pre-/post-clozapine trials

Author (year)	Type of study	Previous Psychotherapy	Number of participants	Reason for clozapine treatment	Dose of clozapine	Outcome Measured	Reported side-effects	Results
Crawford et al. (2022)	Clozapine vs placebo RCT (failure to recruit required number of participants)	Psychotherapy not mentioned	29 (22 female; 7 male)	Resistant to treatment and severe symptoms including high rates of service use and high risk of self-harm	300–400 mg	ZAN-BPD score (multiple secondary outcomes)	Constipation, tachycardia	No statistically significant differences in outcome of clozapine arm compared to placebo arm
Zarzar et al. (2019)	Retrospective cohort-type study without concurrent control	Psychotherapy not mentioned	3 BPD participants out of total 10 participants (all males)	Self-harm (not necessarily diagnosed with BPD but the study included BPD patients)	Median 125 mg daily. Range 75–225 mg daily	Number of episodes of severe self-injurious behaviour measured by visits to urgent care clinic or emergency department	Not reported in results	70% Reduction in visits to urgent care and emergency department after treatment; no statistical analysis
Rohde et al. (2018)	Retrospective cohort-type study without concurrent control	Psychotherapy not mentioned	102 specific BPD patients on clozapine (77 female; 25 male)	N/A (Retrospective review of those who were prescribed clozapine regardless of reason)	Mean 350 mg daily	Psychiatric admissions, psychiatric bed-days, concomitant medications, serious side-effect of clozapine, intentional self-harm, overdose	T2DM (*N*=1)	Statistically significant reduction in hospitalisation, days in hospital, intentional self-harm/overdose, and concomitant medications
White et al. (2017)	Retrospective review without concurrent control	Dialectical behaviour therapy	15 (all females)	Unsatisfactory response to previous pharmacological treatment	Mode 150 mg daily. Range 125–250 mg daily	Incidents of seclusion, restraint and deliberate self-harm; GCAS score	Neutropenia (*N*=1)	Statistically significant reduction in incidents of seclusion, restraint. No statistically significant reduction in self-harm
Dickens et al. (2016)	Retrospective review without concurrent control	Dialectical behaviour therapy	20 (all females)	N/A (any patient treated with clozapine in the designated period)	Dose not mentioned	Subjective clinical experience of participants	Hypersalivation (*N*=11); weight gain (all patients)	Improvement in self-harm, aggression and relationships
Frogley et al. (2013)	Case series	Psychotherapy not mentioned	22 (all females)	High-risk BPD patients engaging in self-harming behaviour	Dose not mentioned	BPRS and GAF scores. Days on enhanced observation, number of therapeutic sessions attended, and count of risk incidents	Weight gain, hypersalivation, tachycardia	Statistically significant improvement in BPRS, aggression, self-directed aggression, number of days on enhanced observations. No significant changes in other outcomes
Fajumi et al. (2012)	Retrospective review without concurrent control	Psychotherapy not mentioned	6 (all females)	Self-injury and restraint	Dose not mentioned	Rates of self-injurious behaviour and incidents of restraint use	Not reported in results	No statistically relevant reduction in self-injury or physical restraint
Haw and Stubbs (2011)	Retrospective review without concurrent control	Psychotherapy not mentioned	30 out of 79 participants on clozapine. Female to male ratio of participants who were prescribed clozapine was not disclosed	N/A (retrospective case note review of already treated patients)	Median 275 mg daily. Range 100–600 mg daily	Subjective clinician assessment of following symptoms: arousal, aggression, self-harm, impulsivity, affective instability and dissociation	Not reported separately for clozapine group	Clozapine more likely to result in major improvement of clinical symptoms compared to other antipsychotics
Swinton (2001)	Retrospective review without concurrent control	Psychotherapy not mentioned	5 (all females)	N/A (retrospective case note review)	400–500 mg daily	Days under observation, and observed self-harm	Not reported in results	Statistically significant decrease in number of days under observation and decrease in number of self-harm events
Chengappa et al. (1999)	Case series; 12 months	Psychotherapy not mentioned	7 (all females)	Self-mutilation and aggression; unsatisfactory response to previous pharmacological treatment	Mean 421 mg daily. Range 300–550 mg daily	Clinical (subjective) and GAF	Sedation, hypersalivation and orthostatic dizziness	Decrease in number of seclusions and restraints. Decrease in injuries to staff and peers. No statistical analysis
Benedetti et al. (1998)	Open-label non-comparative study; 16 weeks	Psychotherapy not mentioned	12 (10 female, 2 male patients)	Psychosis-like symptoms of BPD	25–100 mg daily. Titrated to symptoms	BPRS, HAM-D and GAF	Sedation and decrease in WBC count	Improvement in global function, BPRS scores and HAM-D scores that was statistically significant
Frankenburg and Zanarini (1993)	Open-label non-comparative study; 9 months	Psychotherapy not mentioned	15 (9 female, 6 male patients)	Treatment resistance; BPD with psychotic features	Mean 253 mg daily. Range 70–550 mg daily	BPRS, CGL and GAF	Sedation weight gain, nausea, hypotension-related dizziness	Reduction in BPRS score and symptoms that was statistically significant

### Effect of clozapine pre- and post-treatment within the same patient—single-arm uncontrolled trials

The remaining eleven studies (Zarzar et al. 2019; Rohde et al. 2018; White et al. 2017; Dickens et al. 2016; Frogley et al. 2013; Fajumi et al. 2012; Haw and Stubbs 2011; Swinton 2001; Chengappa et al. 1999; Benedetti et al. 1998; Frankenburg and Zanarini 1993, Table 1) all examined the effects of clozapine before and after its administration (patient presenting as their own control). Two of the studies were prospective studies (Benedetti et al. 1998 and Frankenburg and Zanarini 1993) and the rest were retrospective studies. Several different methods were used to assess the effect of clozapine treatment. Brief Psychiatric Rating Scale (BPRS) score (Overall and Gorham 1962) was used in three studies. One study (Haw and Stubbs 2011) used subjective assessment of improvement by clinicians as an outcome measure. Seven studies used frequency of adverse outcomes (e.g., hospitalisation, self-harm, suicide attempt) before and after treatment as the outcome measure. Seven of the eleven studies conducted statistical analyses of the results and four studies reported descriptive statistics. Out of the seven studies that performed statistical analysis, six studies showed statistically significant improvement post-initiation of clozapine, and one study showed improvement which was not statistically significant. The main adverse drug effects reported from these studies were hypersalivation, sedation, tachycardia, syncope, and metabolic effects. Two studies documented neutropenia (but not agranulocytosis). Four studies did not report adverse effects.

Rohde et al. (2018) conducted the largest study to date for clozapine treatment of BPD. The study utilised the Danish Psychiatric Central Register and identified 102 patients with diagnosis of BPD (without concurrent diagnosis of conditions classically associated with psychosis) who had been trialled on clozapine. This study population represents more than 40% of all the participants in the pre- post- clozapine group. The study found statistically significant reduction in hospitalisation, days in hospital, self-harm, and concomitant use of medications.

### Case reports

There were 12 case reports where clozapine was trialled as a treatment method for BPD (O'Leary et al. 2018; Amamou et al. 2016; Kiani et al. 2015; Argent and Hill 2014; Zarzar and McEvoy 2013; Vohra 2010; Biswas et al. 2008; Rutledge et al. 2007; Biswas et al. 2006; Ferrerri et al. 2004; Steinert et al. 1996; Chengappa et al. 1995, Table 2). Out of these case reports, 10 reported one case each, while Kiani et al. (2015) and Zarzar and McEvoy (2013) reported 4 cases and 5 cases respectively for a total of 20 cases. One of the cases from Kiani et al. (2015) met the exclusion criteria due to concurrent diagnosis of schizophrenia and was removed. In total, 19 cases were included. The rationale for trialling clozapine were ongoing severe BPD-related symptoms despite previous management (*N* = 13), high risk of suicide/self-harm (*N* = 13), aggression (*N* = 6), frequent/multiple admissions (*N* = 2), and impulsivity (*N* = 2); one report did not state the reason. Major symptoms of concern from the reports were self-harm (*N* = 17), aggression (*N* = 12), affective symptoms (*N* = 8), Impulsivity (*N* = 7), anxiety (*N* = 5), pseudohallucinations/hallucinations (*N* = 4), sexual disinhibition (*N* = 1), and compulsions (*N* = 1). All case reports suggested that clozapine may have a positive effect in managing the symptoms of BPD which had not previously responded to multiple other clinical strategies.

**Table 2:** Summary of case reports

Author (year)	Age	Sex	Diagnosis	Major Symptoms	Indication	Other medications tried	Intervention including dose	Length of treatment/follow-up period	Results	Side-effects
O'Leary et al. (2018)	55	Female	BPD	Impulsivity, thoughts of self-harm, affective symptoms, relationship strains	Unsatisfactory response to previous treatment including psychotherapy (CBT and psychodynamic psychotherapy); high risk of suicide; decline in functioning	Amitriptyline, trimipramine, sertraline, escitalopram, fluoxetine, fluvoxamine, duloxetine, venlafaxine, mirtazapine, phenelzine, moclobemide, agomelatine, valproate, lithium, olanzapine, quetiapine.	Clozapine 250 mg daily	2 years follow-up; length of treatment not explicitly stated but presumed to be for the duration of follow-up	Gradual reduction in pervasive thought of suicide and significant improvement on subjective mood. No further suicide attempts.	None mentioned
Amamou et al. (2016)	33	Male	BPD	Repeated suicide attempts and hospitalisations due to impulsivity, irritability, sadness, thoughts of self-harm	Unsatisfactory response to previous treatments including psychotherapy (specifics not mentioned); poor global function; high risk of suicide	Clomipramine, escitalopram, sertraline, paroxetine, venlafaxine, lithium, lamotrigine, olanzapine, risperidone, amilsulpride	Clozapine 300 mg daily + paroxetine 40 mg daily	2 years of treatment and follow-up	Improvement in social functioning and reduction in mood symptoms; reduction in thoughts of self-harm and suicide	None mentioned
Kiani et al. (2015)	36	Female	BPD, mild intellectual disability, depressive disorder and epilepsy	Affective dysregulation Impulsivity/aggression Anxiety Self-injury	Unsatisfactory response to previous pharmacological treatments; aggression and self-harm	Not mentioned	Clozapine dose not mentioned	Not mentioned	Reduction in symptoms; engagement with treatment programmes	None mentioned
Kiani et al. (2015)	37	Female	BPD and mild intellectual disability	Affective dysregulation Impulsivity/aggression Anxiety Self-injury	Unsatisfactory response to previous pharmacological treatments; multiple hospital admissions; aggression and self-harm	Olanzapine, zuclopenthixol, chlorpromazine, amisulpiride, paroxetine	Clozapine dose not mentioned	Not mentioned	No clear outcome reported. States the patient “responded well”	None mentioned
Kiani et al. (2015)	32	Female	BPD, DPD and mild intellectual disability	Affective dysregulation Impulsivity/aggression Anxiety Self-injury, suicide attempts	Unsatisfactory response to previous pharmacological treatments and high levels of impulsivity	Carbamazepine, lithium, fluoxetine, trazodone, sertraline, paroxetine, diazepam, lorazepam, propranolol, zuclopenthixol, flupentixol, thioridazine, risperidone, olanzapine, sulpiride, amisulpriride, droperidol, haloperidol	Clozapine dose not mentioned	Not mentioned	significant reduction in impulsivity and emotional lability; mood remained euthymic	None mentioned
Kiani et al. (2015)	48	Female	BPD and mild intellectual disability	Affective dysregulation Impulsivity/aggression Anxiety, pseudohallucinations Self-harm, suicide attempts	Unsatisfactory response to previous pharmacological treatments; self-harm, suicide attempts and aggression	Antidepressant (not specified), typical antipsychotic (not specified), lorazepam	Clozapine dose not mentioned	4 years follow-up	Stable without serious aggression, self-harm or suicide attempts	None mentioned
Argent and Hill (2014)	17	Female	BPD	Suicide attempts Attempts to harm others Pseudohallucinations	Unsatisfactory response to previous treatments including psychotherapy (DBT and family therapy); high risk to self and others	Risperidone, zuclopenthixol, olanzapine, valproate, promethazine, methylphenidate	Clozapine dose not mentioned	13-week follow-up	Rate of abuse of staff decreased; rate of self-harm decreased. Incidence of challenging behaviour remained similar. Able to be discharged from high care	Constipation, slurring of speech, hypersalivation, weight gain, sedation and tachycardia
Zarzar and McEvoy (2013)	27	Female	BPD	Suicide attempt, self-harm and aggression	Persistent high risk of suicide	Not mentioned	Clozapine 150 mg daily	3 weeks of clozapine followed by 4 weeks off clozapine for total 7 weeks	Marked reduction in self-harm and aggression and no readmission to the hospital; decreased subjective mood disturbance, anxiety and suicidal ideation	None mentioned
Zarzar and McEvoy (2013)	41	Female	BPD	Self-harm and aggression	Frequent use of restraints due to persistent aggression and self-harm	Antipsychotics, antidepressants, and anti-anxiety medications (none specified)	Clozapine 400 mg daily	Not mentioned	Decrease in levels of agitation and aggression; no readmission to the hospital after discharge	None mentioned
Zarzar and McEvoy (2013)	32	Female	BPD	Suicide attempts	Long history of cutting, ongoing suicidal ideation and frequent hospitalisation	Not mentioned	Clozapine 200 mg daily	3 weeks	Reduction in self-injurious episodes and improvement in mood; no readmission to the hospital for 8 months following discharge	None mentioned
Zarzar and McEvoy (2013)	27	Female	BPD	Suicidal ideation and self-harming behaviour	Persistent suicidal ideation and self-harming behaviour	Not mentioned	Clozapine 200 mg daily	5 Weeks	Cessation of suicidal ideation and self-harm; improvement in mood and no readmission	None mentioned
Vohra (2010)	24	Female	BPD	Verbal and physical abuse Self-harm	Unsatisfactory response to previous treatments including psychotherapy (specifics not mentioned); self-harm and aggression	Antidepressants, antipsychotics (not specified	Clozapine 12.5 to 175 mg daily	10 weeks	Improved mood, reduced frequency and severity of self-harm. Improvement in relationships	Weight gain
Biswas et al. (2008)	34	Female	BPD and velocardiofacial syndrome	Self-harm Auditory hallucinations Depressive symptoms Sexual disinhibition	Unsatisfactory response to previous pharmacological treatment	Olanzapine, paroxetine, chlorpromazine, flupenthixol, amisulpride	Clozapine dose not mentioned	Not mentioned	No further admission to hospital. Cessation of self-harm. Marked reduction in psychotic symptoms, maintaining stable relationship	Hypersalivation
Rutledge et al. (2007)	36	Female	BPD	Self-harm, aggression	Not provided	Not mentioned	Clozapine variable dose	2 years	Reduction in number of seclusion but not complete rehabilitation	None mentioned
Biswas et al. (2006)	29	Female	BPD and mild intellectual disability	Self-harm Physical aggression Damage to property High impulsivity, frequent hospital admissions	Unsatisfactory response to previous pharmacological treatment; high impulsivity	Carbamazepine, lithium, fluoxetine, trazodone, sertraline, paroxetine, fluvoxamine, diazepam, propranolol, lorazepam, zuclopenthixol, flupentixol, thioridazine, risperidone, olanzapine, sulpiride, amisulpride, droperidol, haloperidol	Clozapine 12.5 mg BD initially titrated up to 50 mg BD	4 years	Marked reduction in self-harm, physical aggression, damage to property and hospital admissions	T2DM
Ferrerri et al. (2004)	19	Female	BPD	Anxiety, self-mutilating behaviour, suicidal behaviour	Unsatisfactory response to previous pharmacological treatments including psychotherapy; severe self-harm	Paroxetine, clorazepate, cyamemazine, venlafaxine, fluoxetine, valpromide, carbamazepine, diazepam, loxapine, levomepromazine, propericiazine	Clozapine 300 mg daily	4 weeks	Self-mutilating behaviours ceased; patient expressed relief from anxiety symptoms	None mentioned
Steinert et al. (1996)	28	Female	BPD and OCD	Compulsive handwashing; compulsion of control; elaborate dressing and undressing rituals; obsession with sexual content; compulsive aggression	Unsatisfactory response to previous treatments including psychotherapy (family therapy and behavioural therapy)	OCD treatment (not specified), lomipramine, paroxetine, remoxipride, haloperidol, cuclopenthixol	Clozapine 12.5 mg daily initially; titrated up to 400 mg daily	Not mentioned	Reduction in aggression and obsessive sexual content	None mentioned
Chengappa et al. (1995)	32	Female	BPD	Self-mutilating behaviour, aggression, affective instability, hallucinations	Unsatisfactory response to previous pharmacological treatment; self-harm	Chlorpromazine, thioridzine, mesoridazine, loxitane	Clozapine 300 mg daily + chlorpromazine 600 mg daily + lorazepam 3 mg daily	2 months	Dramatic decrease in self-destructive behaviour	None mentioned

### Average Clozapine dose

Excluding case reports, the mean dose of all RCT and single-arm uncontrolled studies was 272 mg daily. The clozapine dose used in the case reports ranged from 25 to 550 mg. Out of the 11 case reports that reported the dose of clozapine used, the mean dose was 252 mg daily. These clozapine doses are on the lower end of the usual dose used for schizophrenia, which ranges between 200 and 600 mg daily (Australian Medical Handbook 2022).

### Risk of bias assessment

The risk assessment of the CALMED study (Crawford et al. 2022) was performed using the RoB 2.0 tool. It was determined that the study is of low risk of bias in all five domains (Table 3).

**Table 3:** RoB 2.0 Risk of bias assessment of included RCT

Study (year)	Bias arising from the randomisation process	Bias due to deviations from the intended intervention	Missing outcome data	Bias measurement of the outcome	Bias in selection of reported result	Overall risk of bias
Crawford et al. 2022	Low risk of bias	Low risk of bias	Low risk of bias	Low risk of bias	Low risk of bias	Low risk of bias

Risk assessment for the remaining non-randomised intervention studies were attempted using the ROBINS-I tool. However, given that none of these studies were true cohort studies, all performed poorly and were at moderate risks of bias (Table 4). In general, retrospective studies performed poorly on all domains except for the “risk of selection of the reported result” domain. The “risk due to confounding” domain specifically was deemed as “serious risk of bias” for all non-RCT studies due to the lack of concurrent controls in any of the non-RCT studies. Out of the remaining five domains, the retrospective studies performed worse than the prospective studies and case series in all the domains except for the “bias in measurement of outcomes” domain. For this domain, the case series had the worst results due to the chance of outcome measures having been influenced by the knowledge of the intervention received.

**Table 4:** ROBINS-I Risk of bias assessment of included non-RCT trials excluding case reports

Study (year)	Bias due to confounding	Bias in selection of participants into the study	Bias in classification of interventions	Bias due to deviations from intended interventions	Bias due to missing data	Bias in measurement of outcomes	Bias in selection of reported result	Overall bias
Zarzar et al. 2019	Serious risk	Moderate risk	Serious risk	Moderate risk	Moderate risk	Serious risk	Low risk	Moderate risk
Rohde et al. 2018	Moderate risk	Moderate risk	Serious risk	Low risk	Low risk	Moderate risk	Low risk	Moderate risk
White et al. 2017	Serious risk	Moderate risk	Serious risk	Moderate risk	Moderate risk	Serious risk	Low risk	Moderate risk
Dickens et al. 2016	Serious risk	Moderate risk	Serious risk	Moderate risk	Moderate risk	Serious risk	Low risk	Moderate risk
Frogley et al. 2013	Serious risk	Serious risk	Moderate risk	Low risk	Low risk	Serious risk	Low risk	Moderate risk
Fajumi et al. 2012	Serious risk	Moderate risk	Serious risk	Moderate risk	Moderate risk	Serious risk	Low risk	Moderate risk
Haw and Stubbs 2011	Serious risk	Moderate risk	Serious risk	Moderate risk	Moderate risk	Serious risk	Low risk	Moderate risk
Swinton 2001	Serious risk	Moderate risk	Serious risk	Moderate risk	Moderate risk	Serious risk	Low risk	Moderate risk
Chengappa et al. 1999	Serious risk	Serious risk	Moderate risk	Low risk	Low risk	Serious risk	Low risk	Moderate risk
Benedetti et al. 1998	Serious risk	Moderate risk	Moderate risk	Low risk	Low risk	Low risk	Low risk	Low risk
Frankenburg and Zanarini 1993	Serious risk	Moderate risk	Moderate risk	Low risk	Low risk	Low risk	Low risk	Low risk

### Case reports quality assessment

The overall quality of the case reports was acceptable. The average number of satisfactory domains in the CARE guideline was 9.00 with the standard deviation of 1.63 (range = 6–12; median = 9) (Table 5).

**Table 5:** Summary of case report quality as per CAse REport (CARE) guidelines checklist

Author (year)	Title	Keywords	Abstract	Introduction	Patient information	Clinical findings	Timeline	Diagnostic assessment	Therapeutic intervention	Follow-up and outcomes	Discussion	Patient perspective	Informed consent
O'Leary et al. (2018)	Sat	Unsat	Unsat	Sat	Sat	Sat	Sat	Unsat	Sat	Sat	Sat	Unsat	Sat
Amamou et al. (2016)	Sat	Sat	Sat	Sat	Sat	Sat	Sat	Unsat	Sat	Unsat	Sat	Unsat	Sat
Kiani et al. (2015)	Unsat	Sat	Sat	Sat	Sat	Sat	Sat	Unsat	Sat	Sat	Sat	Unsat	Sat
Argent and Hill (2014)	Unsat	Sat	Sat	Sat	Sat	Sat	Sat	Unsat	Sat	Sat	Sat	Sat	Sat
Zarzar and McEvoy (2013)	Unsat	Sat	Sat	Sat	Sat	Sat	Sat	Unsat	Sat	Unsat	Equi	Unsat	Unsat
Vohra 2010	Unsat	Sat	Equi	Sat	Sat	Sat	Sat	Unsat	Sat	Sat	Sat	Sat	Sat
Biswas et al. 2008	Sat	Sat	Sat	Unsat	Sat	Sat	Sat	Unsat	Sat	Sat	Equi	Unsat	Sat
Rutledge et al. 2007	Unsat	Sat	Sat	Sat	Sat	Sat	Sat	Unsat	Sat	Unsat	Sat	Unsat	Unsat
Biswas et al. 2006	Sat	Sat	Sat	Sat	Sat	Sat	Sat	Sat	Sat	Sat	Sat	Unsat	Sat
Ferrerri et al. 2004	Unsat	Sat	Sat	Sat	Sat	Sat	Sat	Unsat	Sat	Unsat	Sat	Unsat	Unsat
Steinert et al. 1996	Sat	Unsat	Equi	Unsat	Sat	Sat	Sat	Unsat	Sat	Unsat	Sat	Unsat	Unsat
Chengappa et al. 1995	Unsat	Unsat	Equi	Sat	Sat	Sat	Sat	Sat	Sat	Unsat	Sat	Unsat	Sat

*Sat* satisfactory, *Unsat* unsatisfactory, *Equi* equivocal

## Discussion

Clozapine may have a role in the treatment of a highly selected group of patients with very severe treatment-refractory BPD. Most papers (*n*=18 out of 24 total) in the current review observed benefits from clozapine for patients with BPD that experienced high suicide risk and frequent hospitalisations. However, the majority of included research studies and case reports had significant risk of bias.

A recent non-systematic review by Beri and Boydell (2014) concluded that clozapine potentially has benefits on multiple outcomes of BPD, namely psychotic symptoms, impulsivity, self-mutilation, number of days on enhanced observation, use of restraint, and overall functioning. We have identified two major studies that add substantively to the evidence base, since the review by Beri and Boydell (2014): CALMED RCT (Crawford et al. 2022) and the large retrospective study by Rohde et al. (2018). Concordant with Beri and Boydell (2014), our systematic narrative review, including these two new studies, indicates there is a potential role for clozapine in treating severe treatment-resistant BPD.

### Controlled trials and related research

The only controlled trial performed was the CALMED trial, which was conducted in the UK (Crawford et al. 2022). This trial concluded prematurely due to difficulty recruiting intended numbers. The COVID-19 pandemic was a major reason for premature conclusion of the study. The study also reported two other major difficulties in recruiting participants for the study. Clinicians working in general (non-specialised) wards were reluctant to refer patients to the trial due to their inexperience working with clozapine, and concerns about the high side-effect burden of clozapine. Conversely, clinicians in highly specialised wards were reluctant to refer their patients as they had observed the benefit of clozapine when used for severe BPD and were therefore reluctant to involve high-risk BPD patients on a trial that may involve placebo prescription. Similar barriers for clozapine commencement also occur for those with treatment-resistant schizophrenia, despite gold-standard RCT evidence for clozapine working in this population (Zheng et al. 2022). The CALMED trial used the ZAN-BPD scoring system (Zanarini 2003) as the main outcome measure and did not focus on suicide or self-harm. The results of the trial showed some improvements in ZAN-BPD scores from clozapine use, but these improvements were not statistically significant compared to the placebo arm. This result, however, should be interpreted with caution as the study both failed to recruit required number of participants, and the outcome measure used did not directly address suicide or self-harm.

The study also revealed an interesting insight to the use clozapine in BPD patients. Despite the lack of published evidence, clinicians in very specialised settings in the UK seem to use clozapine in severe BPD. A further UK-based cross-sectional study by Stone-Brown et al. identified that in high-secure hospital settings, up to 40% of severe personality-disorder patients on antipsychotics were on clozapine (Stone-Brown et al. 2016).

### Non-controlled studies

Of the 11 pre-/post-clozapine single-arm uncontrolled trials, 9 were retrospective studies and 2 were prospective studies. In contrast to the CALMED RCT, studies in the pre-/post-clozapine group focused on outcome measures associated with severe BPD. Both prospective studies were conducted before 2000 and there has not been a prospective study since (Benedetti et al. 1998, Frankenburg and Zanarini 1993). Both prospective studies focused on treating psychosis-like symptoms associated with BPD and both found statistically significant benefits from clozapine use.

Of the retrospective studies, 5 performed statistical analyses of the results. Of these, 4 showed statistically significant benefits from clozapine use and 1 did not show statistically significant benefits. Benefits were most commonly seen in reduction of self-harm, levels of aggression, and an improvement in global functioning. All 5 retrospective studies focused on the effect of clozapine on self-harm. Of these 5 studies, 4 showed statistically significant benefit in reducing self-harm or suicide attempts with the use of clozapine, while 1 study did not. Of 5 retrospective studies that did not perform a statistical analysis, all described unquantified benefits from clozapine use.

Rohde et al. (2018) accounted for more than 40% of all participants in the single-arm uncontrolled clozapine group. This study found statistically significant reduction in both hospital use and self-harm. The study performed in Denmark and identified participants using the Danish Psychiatric Central Research Register. This provides evidence there may be benefits from clozapine use for BPD in naturalistic settings. Taking these features of the study by Rohde et al. into account, combined with the fact that the CALMED trial did not focus on reduction of self-harm as the primary outcome, Rohde et al. more specifically addresses the potential effect of clozapine on severe BPD patients at high risk of significant self-harm. One weakness of this trial is its single-arm uncontrolled nature, and thus represents a lower level of evidence than a preferable randomised controlled trial.

The mean clozapine dose used in the non-controlled studies was 265 mg which is lower than the dose of clozapine used for schizophrenia.

### Case reports

Case reports or case series were overwhelmingly favourable regarding outcomes of using clozapine in people with severe BPD who had experienced refractoriness to treatment to both psychotherapy and multiple pharmacotherapy trials. The 12 studies reported 19 cases, and all 19 showed benefits from clozapine use. Out of these 19 cases, 13 quoted suicide risk or self-harm a rationale for trialling clozapine and six of the cases listed aggression as a reason for trialling clozapine. There is a high risk of bias in this group due to the nature of case reports, and these reports must therefore be interpreted in this light. However, such level of overwhelmingly positive results, even from a group of papers with high risk of bias, cannot be ignored. Similar to the non-controlled studies, the dosage of clozapine used was slightly lower (average dose 252 mg) than the doses used for patients with schizophrenia.

### Strengths and limitations

To the best of our knowledge, this is the second review of evidence conducted for the use of clozapine in BPD after the Beri and Boydell study in 2014, and the first systematic narrative review.

The most obvious limitation is the lack of high-quality evidence pertaining to this topic. There are no well-designed and completed RCTs regarding the use of clozapine in severe BPD at risk of suicide. The CALMED randomised trial failed to recruit enough participants, and was terminated early, so did not show a statistically significant improvement from the use of clozapine compared to placebo. Case reports are more supportive but are particularly vulnerable to bias.

There is also a risk of bias in interpreting the various studies included in this systematic narrative review. All included studies did not perform well in the risk of bias assessment, except for the CALMED study, which was determined to be at low risk of bias. The pre-/post-clozapine, uncontrolled trial group mainly were at “moderate risk of bias” on the ROBINS-I risk of bias assessment tool, with only two studies scoring “low risk of bias” (Table 4). For the case reports group, a risk of bias assessment was not performed. However, due to the nature of case reports, there is an inherently high risk of bias notwithstanding their high quality according to CARE guidelines with 9 out of 13 criteria being satisfied on average (Table 5).

The specific clinical models of past psychotherapy treatment were not well described in the reviewed studies. This necessarily limits our interpretation of treatment response and resistance.

Another limitation related to this is the lack of statistical analyses in some studies. There are also a low number of published studies, which increases the risk of random errors. Similarly, most studies had a low number of participants, which again would increase the risk of random errors. Concurrent use of clozapine with other psychotropic medications needed to be included due to prevalent polypharmacy in the treatment of very severe BPD. Ideally, such confounding factors need to be excluded. Articles published in languages other than English were not included in the study. If any relevant articles with different data were published in a language other than English, such data would not have been accessible.

## Conclusion

Clozapine is sometimes used for the treatment of severe treatment-refractory BPD with risk of suicide in both general (Crawford et al. 2022) and forensic settings (Stone-Brown et al. 2016). The available studies provide some support for this clinical practice. More research is needed with well-designed RCTs and prospective studies to determine if there is demonstrable efficacy of clozapine on suicidality and impulsivity for those with severe BPD refractory to psychotherapy and other pharmacotherapy trials. However, it is unlikely that many such trials will be conducted due to the inherent complexity of conducting such a study. Furthermore, as such research involves those at risk of suicide, ethical considerations would make it impractical to conduct a RCT. Accordingly, a retrospective observational study similar to that of Rohde et al. (2018) may be more practicable, despite not being a RCT. A retrospective cohort study comparing patients with BPD who have received clozapine to those who have not will improve the evidence-base. There is preliminary evidence suggesting the efficacy of clozapine in reducing self-harm and suicide risk in BPD patients. Perhaps clozapine may be more effective and safer than non-evidence-based polypharmacy for severe BPD. Clozapine might be considered a potential intervention, amongst others, for the small minority of patients with very severe BPD.
